# The effect of impulsive personality traits on prospective memory under different task importance conditions

**DOI:** 10.1186/s40359-024-01815-5

**Published:** 2024-05-31

**Authors:** Yunfei Guo, Jiaqun Gan, Yongxin Li

**Affiliations:** Institute of Psychology and Behavior, Henan University, Kaifeng, China

**Keywords:** Prospective memory, Impulsivity trait, Task importance, Attention

## Abstract

**Background:**

Prospective memory (PM) refers to the ability to perform planned activities in the appropriate future situations. It needs to be planned in advance and processed through multiple stages such as encoding, retention, retrieval, and execution, which usually require more attention resources. Impulsivity trait individuals are usually characterized by lack of consideration, premature expression, excessive risk-taking and easy to make inappropriate reaction, so they are more likely to show disadvantages in PM. Nevertheless, increasing the importance of PM tasks can promote more adequate and effective cue encoding, and encourage individuals to devote attention to PM tasks, which may change the disadvantage of impulsivity individuals in PM performance.

**Methods:**

In this study, the between-subjects design of 2 (trait type: high-impulsivity trait, low-impulsivity trait) ×2 (task importance: important, unimportant) was adopted in the experiment, the 2-back task was used for the ongoing task, and the focal cues were used for the PM task cues.

**Results:**

The results showed that the PM accuracy of high-impulsivity trait individuals was lower than that of low-impulsivity trait individuals under the task unimportant condition, but there was no difference between the two traits groups under the task important condition.

**Conclusions:**

The results of this study suggested that high-impulsivity trait individuals had a deficit in PM performance, but emphasizing the importance of PM tasks can compensate for their disadvantage in PM performance.

## Introduction

Impulsiveness is a complex and multi-dimensional personality trait. Individuals with impulsivity trait are usually characterized by lack of consideration, premature expression, excessive risk-taking and more prone to inappropriate behavior [1, 2]. Impulsivity trait individuals often fail to adequately and effectively process the events in daily life, which often leads to negative consequences. For example, when driving through an intersection, turning without fully confirming the road conditions is easy to cause traffic accidents. Therefore, the study of impulsivity trait individuals has great practical implications. Early on, Eysenck focused on the impulsive personality in the introversion-extroversion dimension of his Eysenck personality inventory [3]. Since then, a considerable number of studies have focused specifically on the impulsive personality traits and developed tools for the assessment of impulsiveness, of which the most widely used is Barratt impulsiveness Scale-11 (BIS-11) [4]. Barratt and his colleagues divided impulsivity traits into three dimensions: non-planning impulsiveness, attentional impulsiveness, and motor impulsiveness [5]. Non-planning impulsiveness refers to a lack of forethought, not detailed planning and adequate thinking. Attentional impulsiveness refers to the difficulty of focusing on the task being performed and the inability to fully process the task. Motor impulsiveness refers to rapid action without adequately collecting and analyzing information before responding to a task [6, 7]. Impulsivity trait individuals with deficits in these three aspects lead to deficits in a variety of social cognitive abilities, including prospective memory. Deficits in these three aspects of impulsivity trait individuals lead to their impairment in a variety of social cognitive abilities, including prospective memory.

Prospective memory (PM) refers to the ability to perform pre-planned activities in suitable future situations [8]. PM tasks are future-oriented tasks that need to be planned in advance, and inadequate planning will reduce PM performance [9]. Moreover, in most cases, the maintenance of PM intentions and the monitoring of cues require certain attention [10]. When attention resources are insufficient, it is difficult for individuals to effectively monitor cues and maintain PM intentions, thus putting them at a disadvantage in PM performance. In addition, PM studies in laboratory context mostly adopt the dual-task paradigm, which embeds PM tasks into ongoing tasks to simulate the coexistence of ongoing tasks currently being performed and future PM tasks to be performed in real life [11]. The ongoing task, as the dominant task, needs to be performed throughout the experiment and is often regarded as a priority advantageous task, while the PM task, as the secondary task, is usually easily ignored [12, 13]. When encountering PM cues, impulsive responses will make individuals more likely to respond to PM stimuli as ongoing stimuli, resulting in PM targets omission. Therefore, the disadvantage of impulsivity trait individuals in non-planning impulsiveness, attentional impulsiveness, and motor impulsiveness may impair their PM performance.

The available evidence partially supports this point. For example, studies using BIS-11 and PM questionnaires to explore the relationship between impulsiveness and PM performance found that individuals with lower impulsivity scores had better self-reported PM performance [14, 15], which is consistent with our predicitions. However, the use of self-reported questionnaires to reflect an individual’s PM ability tends to be more subjective, while the dual-task paradigm used to measure an individual’s PM ability under the laboratory condition has been widely shown to be objective and effective [16, 17]. Other studies comparing the differences in PM performance between high-impulsivity trait and low-impulsivity trait individuals under the laboratory condition did not find a significant correlation between impulsivity traits and PM performance [18, 19]. Further analysis of these two studies under laboratory conditions showed that the ongoing tasks they used were to ask participants to listen to the recordings and fill out the questionnaire respectively. Such ongoing tasks were relatively simple, and participants still reserved enough attention resources for PM tasks processing when performing the ongoing tasks.

The disadvantages of high-impulsivity trait individuals are closely related to attention in many aspects. First, adequate and effective planning helps individuals to encode tasks more completely, thereby reducing the attention consumption of PM tasks. Effective encoding strategies in the encoding stage can strengthen the connection between PM intentions and behaviors [20], while longer and more adequate encoding will also improve individual familiarity with tasks, making PM intentions easier to be retrieved by individuals [21, 22], all of which can reduce the dependence of tasks on attention. High-impulsivity trait individuals have defects in non-planning impulsiveness, which makes it difficult for them to improve the automatic processing level of PM tasks through effective and deep encoding during the encoding stage. Secondly, reducing the behavioral response speed can allow individuals to reserve more attention resources for PM tasks processing, especially when they encounter PM cues, slowing down the response speed is more conducive to them to occupy more attention for PM cues recognition, intentions retrieval and tasks switching [23, 24]. Due to their deficiency in motor impulsiveness, individuals with high-impulsivity trait do not have enough attention resources for deeper processing of stimuli before responding, and they are prone to quickly make wrong ongoing responses when encountering PM cues. Thus, high-impulsivity trait individuals have inherent deficits in attention, which are more likely to manifest easily under the high attention load condition. Therefore, the first purpose of this study was to test whether high-impulsivity trait individuals would perform worse in PM performance compared to low-impulsivity trait individuals under the difficult ongoing task condition.

PM performance in high-impulsivity trait individuals may be greatly affected by task importance. In the typical dual-task paradigm, PM tasks are usually regarded as subsidiary tasks, while ongoing tasks are usually performed as priority tasks [12, 13]. However, in real life, individuals usually plan something important as the focus of their work for the day. For example, remember to take your insulin after meals, and remember to hand in your project application when passing by the social science office. When encountering these important PM tasks, individuals’ attitude towards the processing of PM tasks also change. The motivational cognitive model (MCM) believes that increasing the importance of tasks mainly improves an individual’s PM performance in two ways. The first is to promote the automated processing level of PM through the use of some strategies in the encoding stage [25], which can compensate for the disadvantage of high-impulsivity trait individuals in non-planning impulsiveness. The second is that individuals in the maintenance and retrieval stages of PM improve the attention resources available for PM tasks at the cost of slowing down the response speed of tasks and reducing the performance of ongoing tasks, so that PM tasks can be more fully processed [26, 27]. Therefore, task importance may compensate for the disadvantage of high-impulsivity trait individuals in attentional impulsiveness and motor impulsiveness, thus improving PM performance. The second aim of this study was to examine whether emphasizing task importance could improve PM performance in high-impulsivity trait individuals.

According to the above analysis, we hypothesized that in the task unimportant condition, high-impulsivity trait individuals would have worse PM performance, faster reaction time, and less adequate encoding of PM tasks than low-impulsivity trait individuals. There was no difference in PM performance between the two groups in the task important condition. This study attempts to expand the research scope of the influencing factors of PM in high-impulsivity trait individuals, further clarify the boundary conditions of their disadvantage in PM, and deepen our understanding of their social cognitive ability.

## Method

### Participants

230 participants completed the Barratt Impulsiveness Scale-11 (BIS) [4], questionnaire separately. Participants with a score in the top 27% (total score ≥ 109) were identified as the high-impulsivity trait group. Those who scored in the bottom 27% (total score ≤ 84) were identified as the low-impulsivity trait group. Since participants with the same score can be considered as homogeneous participants, all the participants with a score of 109 will be included in the high-impulsivity trait group, while all the participants with a score of 84 will be included in the low-impulsivity trait group. After excluding the data with a score less than 0.55 in the ongoing task (since the 2-back task was used, the random score of this task was 0.5, so the score less than 0.55 could be considered as too low) and without any PM response, 119 effective participants were finally obtained (*M*_age_=20.63, *SD* = 1.25, *N*_female_=78). All participants were tested individually. They were informed about the content and procedure of the experiment and signed an informed consent form before the experiment. They were paid about $5 if they successfully completed the experiment. This study was approved by the Institutional Review Board of Henan Provincial Key Laboratory of Psychology and Behavior.

### Experimental design

A between-subjects design of 2 (trait type: high-impulsivity trait, low-impulsivity trait) ×2 (task importance: important, unimportant) was used in this study.

### Experimental tasks and procedures

At the beginning of the experiment, specific requirements for ongoing task and PM task were presented. The ongoing task was a 2-back task, which required participants to do not need to react in the first two letters of the program, starting from the third letter and then comparing it with the second letter in front of it in turn (the third letter is compared with the first letter, and the fourth letter is compared with the second letter…). When the two letters were the same, the participant needed to press the J key, otherwise they should press the F key. The time from the presentation of instructions to the appearance of experimental stimuli was recorded by the experimenter and counted as the time of the encoding stage. At the beginning of each ongoing task, a “+” fixation point was displayed for 300 milliseconds, followed by an English capital letter, which disappeared after the participant responded. The letter was displayed for a maximum of 2000 milliseconds, and finally a blank screen was displayed for 300 milliseconds. The PM task required participants to press the space bar directly when encountering either G or R, without performing the letter comparison response. In the task important condition, participants were additionally told that the PM task was the focus of our attention and was an important task. In the task unimportant condition, participants were not told whether the task was important or not. The procedure was the same in all conditions, containing 150 ongoing trials, of which 10 PM trials were inserted, with at least 10 ongoing trials spaced between each two PM trials.

## Results

### Prospective memory accuracy

**Fig. 1:**
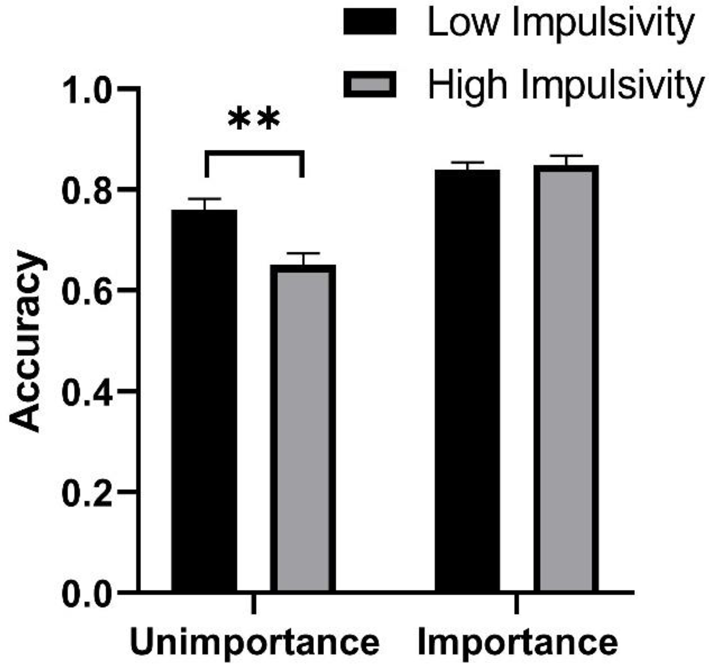
The prospective memory accuracy of low impulsivity trait group and high impulsivity group in different task importance conditions. Two asterisks represent *p* < 0.01

The results of ANOVA showed that the main effects of trait type was significant, *F* (1, 115) = 6.48, *p* < 0.05, η_p_^2^ = 0.05, and the main effects of task importance was also significant, *F* (1, 115) = 51.16, *p* < 0.001, η_p_^2^ = 0.31. There was a significant interaction effect between trait type and task importance, *F* (1, 115) = 7.42, *p* < 0.01, η_p_^2^ = 0.06. A further simple effect showed that in the task unimportant condition, the low-impulsivity trait group had higher PM accuracy than the high-impulsivity trait group, *p* < 0.01, but there was no difference in PM accuracy between the two trait groups under the task important condition. (see Fig. 1; Table 1).

**Table 1:** The accuracy and reaction time of prospective memory

		PM Accuracy	PM Reaction Time (milliseconds)
Unimportant Condition	Low Impulsivity	0.76 (0.12)	939 (178)
	High Impulsivity	0.65 (0.13)	834 (188)
Important Condition	Low Impulsivity	0.84 (0.08)	980 (190)
	High Impulsivity	0.85 (0.10)	956 (137)

### Prospective memory reaction time

**Fig. 2:**
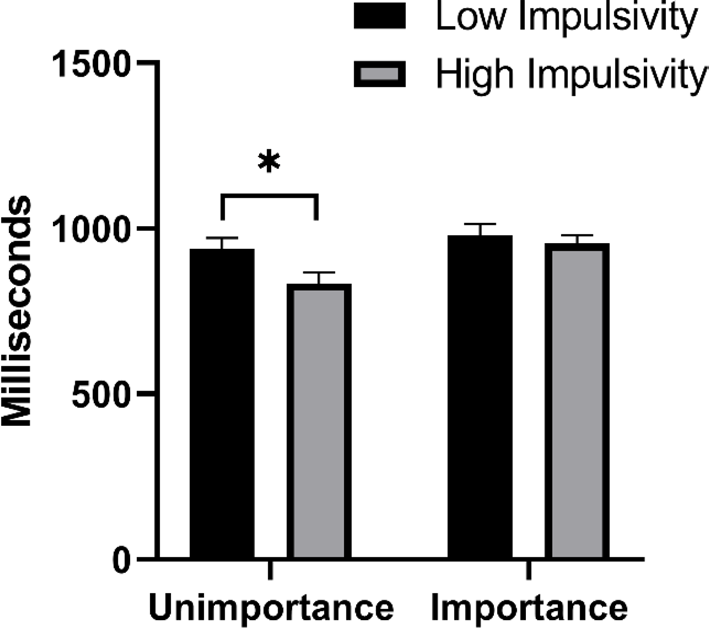
The prospective memory reaction time of low impulsivity trait group and high impulsivity group in different task importance conditions. An asterisk represents *p* < 0.05

The results of ANOVA showed that the main effect of task importance was significant, *F* (1, 115) = 6.58, *p* < 0.05, η_p_^2^ = 0.05, the PM reaction speed of the task important condition was slower than that of the task unimportant condition. The main effect of trait type was significant, *F* (1, 115) = 4.14, *p* < 0.05, η_p_^2^ = 0.04, the reaction speed of the high-impulsivity trait group was faster than that of the low-impulsivity trait group. The interaction between task importance and trait type was not significant, *p* > 0.05. We focused on whether there are differences between individuals with low impulsivity and individuals with high impulsivity under different task importance conditions, but we did not find significant interaction effects, so we could not further conduct a simple effect test. To analyze the results more specifically, we used a t-test to compare differences between the two trait groups under the task important and unimportant conditions. The results showed that under the task unimportant condition, the response speed of the high-impulsivity trait group was significantly faster than that of the low-impulsivity trait group, *t* (57) = 2.21, *p* < 0.05, Cohen’s *d* = 0.57. However, under the task important condition, there was no difference in response speed between the high-impulsivity trait group and the low-impulsivity trait group, *p* > 0.05 (see Table 1; Fig. 2).

It is worth pointing out that t-test was used to separately analyze the differences between different trait type groups under the two task importance conditions, which can provide more information to reveal the differences in PM performance and processing mechanism between different trait type groups. However, this method of using t-test analysis alone may slightly increase the risk of type II error. Therefore, when a significant difference was found through the t-test, we would not regard the difference as a deterministic conclusion, but as an auxiliary explanation with some basis. In the analysis of the following indicators, if no interaction effects were found in the results of ANOVA, we would still further use t-test to analyze the results of our interest.

### Ongoing task accuracy

The results of ANOVA showed that the main effect of task importance was significant, *F* (1, 115) = 5.90, *p* < 0.05, η_p_^2^ = 0.05, and the accuracy under the task important condition was higher than that under the task unimportant condition. The main effect of trait type was significant, *F* (1, 115) = 6.11, *p* < 0.05, η_p_^2^ = 0.05, the accuracy of the high-impulsivity trait group was lower than that of the low-impulsivity trait group. We further used t-test to compare whether there was any difference between the accuracy of the two trait groups under the task important condition and the task unimportant condition. The results showed that under the task unimportant condition, the accuracy of the high-impulsivity trait group was significantly lower than that of the low-impulsivity trait group, *t* (57) = 2.47, *p* < 0.05, Cohen’s *d* = 0.67. Under the task important condition, there was no difference in the accuracy between the two trait types, *p* > 0.05 (see Table 2).

**Table 2:** The accuracy and reaction time of ongoing task

		Accuracy Ongoing Task	Ongoing Task Reaction Time (milliseconds)
Unimportant Condition	Low Impulsivity	0.84 (0.03)	817 (135)
	High Impulsivity	0.81 (0.05)	744 (125)
Important Condition	Low Impulsivity	0.81 (0.04)	886 (152)
	High Impulsivity	0.80 (0.04)	795 (132)

### Ongoing task reaction time

**Fig. 3:**
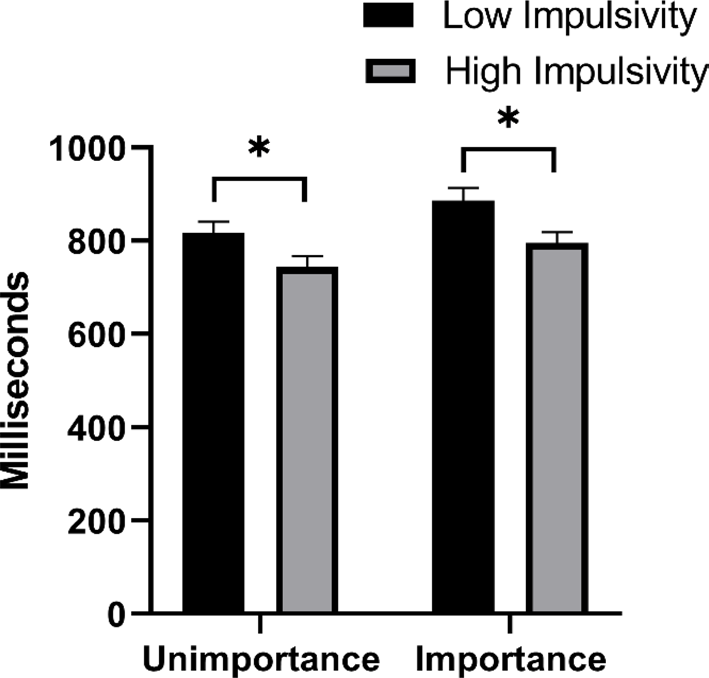
The ongoing task reaction time of low impulsivity trait group and high impulsivity group in different task importance conditions. An asterisk represents *p* < 0.05

The results of ANOVA showed that the main effect of task importance was significant, *F* (1, 115) = 5.73, *p* < 0.05, η_p_^2^ = 0.05, the reaction speed under the task important condition was slower than that under the task unimportant condition. The main effect of trait type was significant, *F* (1, 115) = 10.69, *p* < 0.001, η_p_^2^ = 0.09, and the high-impulsivity group responded significantly faster than the low-impulsivity group. We further used t-test to compare whether there was any difference between the ongoing task reaction time of the two trait groups under the task important and unimportant conditions, and found that the high-impulsivity group responded faster than the low-impulsivity group under the task unimportant condition, *t* (57) = 2.14, *p* < 0.05, Cohen’s *d* = 0.56; Under the task important condition, the high-impulsivity trait group still responded faster than the low-impulsivity trait group, *t* (58) = 2.47, *p* < 0.05, Cohen’s *d* = 0.64 (see Table 2; Fig. 3).

### Number of strategies

The results of ANOVA showed that the main effect of task importance was significant, *F* (1, 115) = 7.37, *p* < 0.01, η_p_^2^ = 0.06. The number of strategies in the task important condition was more than that in the task unimportant condition. We further used t-test to compare whether there was any difference between the strategies of the two trait groups under the task important and unimportant condition, and found that there was no difference in the number of strategies between the two trait type groups under either the task important condition or the task unimportant condition, *ps* > 0.05 (see Table 3).

**Table 3:** The number of strategies and encoding duration

		Number of Strategies	Coding Duration (seconds)
Unimportant Condition	Low Impulsivity	0.47 (0.68)	84.83 (15.36)
	High Impulsivity	0.45 (0.63)	74.83 (17.85)
Important Condition	Low Impulsivity	0.79 (0.73)	88.21 (22.65)
	High Impulsivity	0.84 (0.82)	89.68 (16.89)

### Encoding duration

**Fig. 4:**
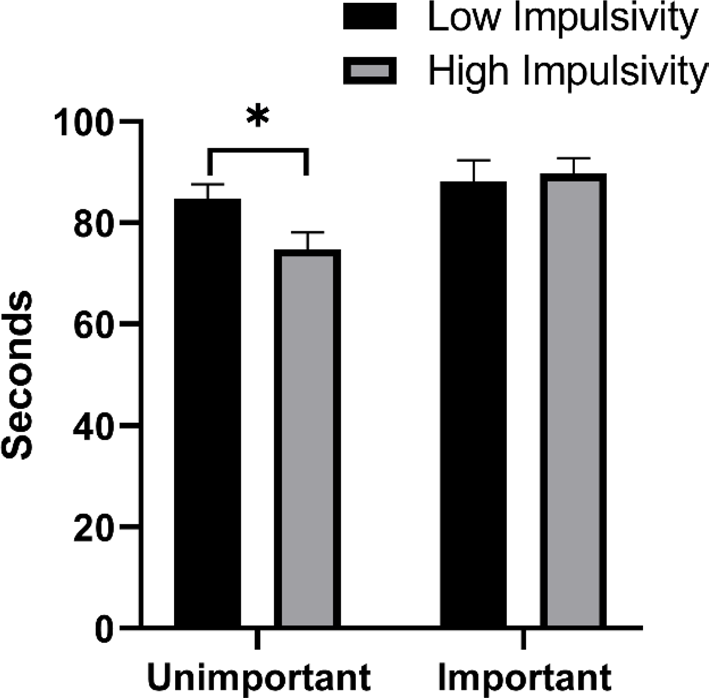
The encoding duration of low impulsivity trait group and high impulsivity group in different task importance conditions. An asterisk represents *p* < 0.05

The results of ANOVA showed that the main effect of task importance was significant, *F* (1, 115) = 8.16, *p* < 0.01, η_p_^2^ = 0.07, the encoding duration under the task important condition was longer than that under the unimportant condition. We further used t-test to compare whether there was a difference between the encoding duration of the two trait groups under the task important and unimportant conditions, and found that the encoding duration of the high-impulsivity group was shorter than that of the low-impulsivity group under the task unimportant condition, *t* (57) = 2.08, *p* < 0.05, Cohen’s *d* = 0.54; Under the task important condition, the encoding duration of the high-impulsivity group was not different from that of the low-impulsivity group, *p* > 0.05 (see Table 3; Fig. 4).

## Discussion

The PM contains multiple stages, including the encoding stage, maintenance stage, retrieval stage, and execution stage, and in most cases requires a lot of self-initiated attention resources [28, 29]. Since impulsivity trait individuals are characterized by non-planning impulsiveness, attentional impulsiveness, and motor impulsiveness, they may not adequately process PM tasks at multiple stages, which may lead to their disadvantage in PM performance. The first aim of this study was to test that whether individuals with impulsive personality were at a disadvantage in PM performance. The results of the PM accuracy showed that the high-impulsivity trait group had lower accuracy than the low-impulsivity trait group under the task unimportant condition. The results validates our hypothesis that individuals with high-impulsivity trait have defects in PM, which is also consistent with the results of some studies [14, 15]. Cuttler et al. [14] and Gladwin et al. [15] both used self-reported questionnaires to measure the PM performance of individuals with different personality traits. Although this method was closer to their PM performance in life, the self-reported results lacked objectivity. Other studies have measured the PM performance in a laboratory setting [18, 19], but their findings were inconsistent with the results of those studies using self-reported questionnaires, as well as with the results of this study. We originally speculated that the reason for the inconsistent results between the laboratory setting and the self-reported questionnaires was that the ongoing task in the laboratory setting was so simple that there was a ceiling effect. Therefore, the current study adopted the more difficult 2-back task as the ongoing task, and found that the PM performance of high-impulsivity trait individuals was defective under the high load condition. Therefore, in a laboratory setting, the inconsistency between the results of our study and other studies should be attributed to differences in the availability of attention resources for PM tasks.

Increasing the importance of PM tasks may compensate for the deficit of high-impulsivity trait individuals in PM performance. According to the MCM, emphasizing the importance of PM tasks prompts individuals to improve PM performance by allocating more attention to PM tasks and by utilizing other methods that facilitate spontaneous retrieval of PM intentions (e.g., adopting effective encoding strategies, conducting more in-depth processing on PM tasks during the encoding stage, etc.) [25, 30]. To some extent, these methods can compensate for the disadvantages of individuals with high-impulsivity trait in the characteristics of non-planning impulsiveness, attentional impulsiveness, and motor impulsiveness, thus improving their PM performance. Our results of PM accuracy showed that the PM performance of both trait type groups was better in the task important condition than in the task unimportant condition, which indicated that the manipulation of task importance in our study was effective. In addition, we found that under the task important condition, there was no difference in PM accuracy between the high-impulsivity trait group and the low-impulsivity trait group, suggesting that the manipulation of task importance can indeed compensate for the impairment in PM performance of high-impulsivity trait individuals.

Why did PM performance in individuals with high-impulsivity trait improve to a greater extent under the task important condition? Before clarifying this question, it is necessary to identify the reasons why individuals with high-impulsivity trait are disadvantaged in PM performance. Given that high-impulsivity trait individuals have deficits in the characteristics of non-planning impulsiveness, attentional impulsiveness, and motor impulsiveness [7, 14], it is reasonable to speculate that their poor PM performance may be due to these defects. This speculation is supported by some findings. Firstly, we found that under the task unimportant condition, the high-impulsivity trait group responded faster to the ongoing task than the low-impulsivity trait group, and the accuracy of ongoing tasks and PM tasks in the high-impulsivity trait group was also significantly lower than that in the low-impulsivity trait group, indicating that the high-impulsivity trait group did not reserve enough attention resources for the processing of ongoing tasks and PM tasks during the PM intention maintenance period. It reflects a deficit in their characteristics of attentional impulsiveness. In addition, compared with the task unimportant condition, the ongoing task in the task important condition had a higher accuracy and a slower reaction time, indicating that emphasizing the importance of PM task would prompt individuals to devote more attention resources to process PM tasks. Thes results validates MCM’s prediction [27]. However, under the task important condition, the ongoing task response speed of the high-impulsivity trait group was still faster than that of the low-impulsivity trait group, which indicated that task importance promoted the attention level of the two trait groups similarly, and did not motivate the high-impulsivity trait group to spend more attention resources to maintain PM intentions to a greater extent. Secondly, this study found that under the task unimportant condition, the high-impulsivity trait group also responded faster to PM targets than the low-impulsivity trait group, suggesting that high-impulsivity trait individuals did not engage too much time for checking and confirming PM targets when encountering PM cues, but tended to respond quickly. This behavior reflects that high-impulsivity trait individuals show the deficit in motor impulsiveness when performing PM tasks. However, under the task important condition, there was no difference between the two trait groups, indicating that task importance may compensate for the disadvantage of high-impulsivity trait individuals in motor impulsiveness characteristics. Finally, we used encoding duration and the number of encoding strategies to examine the non-planning impulsiveness characteristics of impulsivity traits. Encoding duration can reflect the adequacy of encoding content to a certain extent, while encoding strategies can affect the effectiveness of encoding information [20, 31]. In terms of encoding strategies, we found that the task important condition did increase the number of strategies for both trait types, which is consistent with MCM. However, we did not find any difference between the two trait types in both task important and unimportant conditions. Besides, this study found that the encoding duration was shorter in the high-impulsivity trait group than in the low-impulsivity trait group under the task unimportant condition, but there was no difference between the two groups under the task important condition. The results of encoding duration reveal that individuals with high-impulsivity trait are more likely to show inadequacy in task encoding due to their defects in non-planning impulsiveness characteristics, but task importance can compensate for this deficiency to some extent. In conclusion, individuals with high-impulsivity trait do have impairments in the characteristics of non-planning impulsiveness and motor impulsiveness, but task importance can make up for their shortcomings in these two characteristics.

This study preliminarily explored the PM performance of impulsivity traits individuals under different task importance conditions, which to some extent revealed the boundary conditions and processing mechanisms of impulsivity trait individuals’ defects in PM, with certain practical implications. However, there are some limitations to this study. For example, the focal PM cues were used in this study for PM tasks. Focal cues have been shown to be less dependent on attention, and can even achieve spontaneous retrieval of PM intentions under certain conditions [32]. Therefore, even though individuals with high-impulsivity trait still reserved less attention resources under the task important condition, their PM performance did not decrease significantly. However, in the nonfocal cues condition, the successful execution of the PM task must require self-initiated attention resources [33]. In this case, individuals with high-impulsivity trait may struggle to improve their PM performance if they are consistently at a disadvantage in attention. Therefore, future research should further focus on whether PM performance in individuals with impulsivity traits is still affected by task importance in the nonfocul cue condition.

## Conclusions

This study examined the effect of task importance on PM in impulsivity trait individuals. The results showed that individuals with high-impulsivity trait were indeed at a disadvantage in PM performance, but emphasizing the importance of PM tasks could compensate for their deficiencies in PM. This study explores the external factors that affecting the PM performance of impulsivity trait individuals, enhances our understanding of the cognitive ability of this group, and also provides some insights for improving their PM ability in daily life.

## Data Availability

The datasets used during the current study is available upon request to the corresponding author.
